# Fatty Acid Profile and Metabolism Are Related to Human Sperm Parameters and Are Relevant in Idiopathic Infertility and Varicocele

**DOI:** 10.1155/2020/3640450

**Published:** 2020-08-31

**Authors:** Giulia Collodel, Elena Moretti, Daria Noto, Francesca Iacoponi, Cinzia Signorini

**Affiliations:** ^1^Department of Molecular and Developmental Medicine, University of Siena, Siena, Italy; ^2^Department of Food Safety, Nutrition and Veterinary Public Health, National Institute of Health, Rome, Italy

## Abstract

**Objectives:**

Fatty acids (FA) modulate oxidative stress, reactive oxygen species (ROS) production, and inflammatory processes in spermatogenesis.

**Methods:**

The amount of 17 different FAs and the level of F_2_-isoprostanes (F_2_-IsoPs) and cytoplasmic phospholipase A_2_ (cPLA_2_) were compared and correlated to sperm characteristics; these last ones were evaluated by light and electronic microscopy in varicocele and idiopathic infertile patients.

**Results:**

Total n-3 polyunsaturated acids (PUFAs) and docosahexaenoic acid (DHA), one of the n-3 PUFAs, were significantly reduced in idiopathic infertile men compared to controls (*P* < 0.05). In the whole studied population, oleic acid and total monounsaturated acids (MUFAs) correlated negatively with sperm concentration, progressive motility, normal morphology, vitality, and fertility index and positively with sperm necrosis. Eicosapentaenoic acid (EPA) amount was positively correlated with the percentage of sperm necrosis and cPLA_2_ level and negatively with sperm concentration. Sperm vitality was negatively correlated with the saturated fatty acids (SFAs). In infertile groups, cPLA_2_ was negatively correlated with DHA and n-3 PUFAs (both *P* < 0.05) and positively with EPA (*P* < 0.05). In the varicocele group, sperm vitality was negatively correlated with palmitoleic acid and total n-6 PUFAs (*P* < 0.05); sperm apoptosis was positively correlated with the total SFA percentage (*P* < 0.05).

**Conclusions:**

FA composition in sperm membrane and the metabolism of sperm FAs are interrelated parameters, both relevant in sperm maturation processes and fertility.

## 1. Introduction

Phospholipids, glycerophospholipids, and cholesterol define the lipid composition of the plasma membranes [1]; in spermatozoa, it is directly related to sperm physiology regulating cellular osmotic balance [2], sperm motility [3], and acrosome reaction and sperm-oocyte fusion [4]. In animals, the lipid sperm composition is described, and it seems to influence external or internal fertilization in fishes [5], seasonal changes in stallions [6], and the ability to survive the cryopreservation process in different species [7].

Fatty acids (FAs) esterified to phospholipids are part of the cell membrane and contribute to the cell membrane structure. As we all know, FAs are classified, according to the presence of double bonds in their chain structure, as saturated FAs (SFAs), in the absence of double bonds, as monounsaturated FAs (MUFAs), when a single double bond is present, and as polyunsaturated FAs (PUFAs) when having two or more double bonds. In a sperm membrane, FA composition, as a different ratio in PUFAs, MUFAs, and SFAs, seems to be a relevant factor in explaining sperm quality. Cell flow and elasticity of plasma membranes are linked to the presence of PUFAs which are particularly abundant in mammalian spermatozoa. In human sperm, docosahexaenoic acid (DHA, C22:6n-3) and palmitic acid (C16:0) are the predominant PUFAs and SFAs [8], respectively. In addition, it has been reported that sperm motility, morphology, and concentration are positively associated with DHA levels [9, 10], whereas low concentrations of DHA have been observed in asthenozoospermic and oligozoospermic men [3, 11]. A negative correlation between increased SFAs [12] or *trans* FA levels [13] and normal sperm parameters was also described.

As a not secondary issue, the enzymatic, or free radical-induced, PUFA metabolism which influences inflammatory process and susceptibility to lipid peroxidation events in mammalian sperm membrane should be mentioned [4, 14, 15]. In particular, cyclooxygenase (COX) activities on arachidonic acid (ARA) lead to the synthesis of prostaglandins, prostacyclin, and thromboxane A_2_, whereas ARA metabolism by lipoxygenase (LOX) activities generates leukotrienes [15]. On the other hand, eicosapentaenoic acid (EPA) and DHA are converted, by the same enzymes, COX and LOX, to different series of prostanoids and leukotrienes, respectively. Moreover, specialized proresolving mediators (SPM) with potent anti-inflammatory and immunoregulatory actions are derived from enzymatic oxidation of DHA and EPA [16]. With reference to the free radical-induced PUFA metabolism, isoprostanes (IsoPs) and neuroprostanes originate from nonenzymatic PUFA (i.e., ARA, DHA, and EPA) metabolism and they are known as the secondary products of lipid peroxidation and markers of oxidative damage [17, 18]. Since IsoPs are originally formed *in situ*, on FA precursors esterified in phospholipids, and subsequently released from membrane phospholipid as free acids by phospholipase action [17], esterified IsoPs quantified from PUFA cell membrane are the specific index of membrane PUFA oxidation.

On PUFA metabolism, phospholipase activity and isoprostane formation are currently described as key factors in influencing male fertility and sperm membrane stability, capacitation, and immaturity [15, 19–21].

The purpose of this manuscript was to investigate the membrane FA profile in the sperm of patients with different reproductive pathologies. As a relevant novelty in human male fertility, a close relationship between the FA pattern in a sperm membrane and the metabolism of sperm FA has been shown. We carried out comparisons and correlations between the amounts of 17 different FAs and the levels of F_2_-isoprostanes (F_2_-IsoPs) and phospholipase A_2_ (PLA_2_) with sperm characteristics. We reported these last ones evaluated by light and electronic microscopy.

## 2. Materials and Methods

### 2.1. Patients

From September 2018 to April 2019, we selected 24 infertile patients (aged 30 to 40 years) attending our centre for semen analysis. The duration of infertility was in the range 3–6 years of unprotected sexual intercourses without conception.

The inclusion criteria were as follows: nonazoospermic men with a normal karyotype evaluated by conventional cytogenetic analysis, BMI < 25 kg/m^2^ and no history of diabetes, radiotherapy, chemotherapy, chronic illness or medication, neither of the use of drugs, and alcohol and dietary supplements. All the individuals showed normal concentrations of follicle stimulating hormone (FSH), luteinizing hormone (LH), and testosterone (T). The patients declared that they were not professionally exposed to pesticides or heavy metal. Subjects with leukocytospermia, genitourinary infection, and heavy smoking habits (>10 cigarettes/day) were excluded. The presence of varicocele was detected by physical examination and scrotal eco-color Doppler analysis performed in all selected patients. The possible presence of clinically asymptomatic genitourinary infections was investigated by a bacteriological analysis. Patients with positive bacteriological cultures were considered infected and excluded consequently.

The patients enrolled for this study were then divided into two groups according to clinical diagnosis:

- Group I: idiopathic infertility (*n* = 12)
- Group II: varicocele (*n* = 12)—where left sided varicocele was found in 10 cases (grade III in three patients, grade II in five patients, and grade I in two patients) and right sided varicocele and bilateral varicocele were diagnosed in one (grade II) and one (grade I-II), respectively

A group of six fertile men (aged 32-39 years) was used as controls. Men fathered at least one child in the last 3 years. They were not affected by infections, hormonal and anatomical problems.

All subjects were informed with respect to this study, and they provided an informed written consent before the inclusion on this research. The study was approved by the Ethics Committee of Azienda Ospedaliera Universitaria Senese, CEAOUS.

### 2.2. Light Microscopy

Subjects enrolled in this study were asked to abstain from intercourse and masturbation for a period of 3–4 days before the semen collection.

According to the WHO guidelines [22], samples were collected in a bathroom closed to the laboratory, in order to limit the exposure of the semen to fluctuations in temperature and to control the time between collection and analysis. Samples were analyzed after liquefaction for 30 min at 37°C; a normal liquefied semen sample had a homogeneous, grey opalescent appearance. Volume was formerly measured by pipetting the semen into calibrated containers, pH with pH indicator papers in the range 6.0–10.0.

The motility of sperm in a sample volume of 10 *μ*l was evaluated microscopically at 400x magnification. Sperm were classified as progressively motile, locally motile, or immotile. Sperm morphology was assessed by the Papanicolaou (PAP) test modified for spermatozoa. The morphology of 200 spermatozoa was determined under a light microscope at 1,000x magnification using an oil-immersion objective. Sperm vitality was detected using 0.5% eosin Y (CI 45380) in a 0.9% aqueous sodium chloride solution; one hundred stained (dead) cells and unstained (living) cells were scored.

The possible presence of leukocytes was explored by peroxidase stain; a concentration > 1 × 10^6^ cell/ml was considered out of range and identified as leukocytospermia. A sample from each patient was analyzed. After semen evaluation, all the samples were divided in three aliquots to be used for the different analyses.

### 2.3. Transmission Electron Microscopy (TEM)

An aliquot was processed for transmission electron microscopy (TEM). Sperm samples were fixed in a cold Karnovsky fixative and maintained at 4°C for 2 h. Then, the semen was washed in 0.1 mol/l cacodylate buffer (pH 7.2) for 12 h, postfixed in 1% buffered osmium tetroxide for 1 h at 4°C, and washed again in 0.1 mol/l cacodylate buffer; the samples were dehydrated in a graded ethanol series and embedded in Epon Araldite. Ultrathin sections were cut with a Supernova ultramicrotome (Reichert Jung, Vienna, Austria), mounted on copper grids, stained with uranyl acetate and lead citrate, and then observed and photographed with a Philips CM12 transmission electron microscope (TEM; Philips Scientifics, Eindhoven, the Netherlands, Centro di Microscopie Elettroniche “Laura Bonzi,” ICCOM, Consiglio Nazionale delle Ricerche (CNR), Via Madonna del Piano 10, Firenze, Italy).

Three hundred sperm sections were analyzed from each sample. TEM data was elaborated using a mathematical formula [23] which provides numerical scores such as fertility index (number of sperm free of structural defects in a semen sample) and percentage of sperm pathologies such as immaturity, apoptosis, and necrosis [24], defined by distinctive ultrastructural characteristics. Immaturity includes the presence of cytoplasmic droplets, altered acrosomes, and roundish or elliptical nuclei with uncondensed chromatin. Marginated chromatin, translucent vacuoles included in cytoplasmic residues, and swollen and badly assembled mitochondria are the ultrastructural indicators of apoptosis, whereas sperm with reacted or absent acrosome, misshaped nuclei with disrupted chromatin, broken plasma membrane, and poor axonemal and periaxonemal cytoskeletal structures are affected by necrosis.

### 2.4. Analyses of Sperm Membrane Fatty Acid (FA) Composition

An aliquot of each semen sample was centrifuged at 400g for 15 min; then, the supernatant was discarded. Sperm membrane phospholipids were extracted and subsequently converted into fatty acid methyl esters (FAMEs) by a transesterification procedure performed in the presence of a methanol solution of 0.5 M KOH [25]. FAMEs were analyzed by a gas chromatography instrumentation (GC) (Agilent 6850, Milan) equipped with a capillary column (DB23, Agilent, USA) and a flame ionization detector. Hydrogen as carrier gas and FAMEs were identified by comparison with the retention times of authentic molecules [25]. All the determinations were performed by the Lipidomics Laboratory at Lipinutragen Srl, CNR Area della Ricerca di Bologna, Italy.

### 2.5. Phospholipase A_2_ (PLA_2_) Determination

An aliquot of each semen sample was centrifuged at 400 g for 15 min; then, the pellet was discarded. The supernatant was used for cytosolic PLA_2_ (cPLA_2_) and F_2_-isoprostane (F_2_-IsoP) evaluations.

Cytosolic PLA_2_ (cPLA_2_) amounts in seminal plasma samples were determined by an enzyme-linked immunosorbent assay (ELISA, AMSBIO, distributed by D.B.A. Italia). In particular, the determinations were carried out as a competitive immunoassay, where standard samples (known cPLA_2_ amounts) or seminal samples were added (50 *μ*l) to wells precoated with a monoclonal antibody to cPLA_2_. cPLA_2_ is present in the standards, or human samples competed with the biotin-conjugated antigen to bind to the capture antibody. The final revelation was performed using an avidin horseradish peroxidase. The tetrametilbenzidina (TMB) substrate was then added to develop a color changing into yellow after the addition of an acidic stop solution. Spectrometric detection of color intensity at 450 nm allowed the determination of cPLA_2_ amounts by comparing the optical density of the seminal samples to the standard curve (ranging from 16 ng/ml to 0.5 ng/ml cPLA_2_ amounts).

### 2.6. F_2_-Isoprostane (F_2_-IsoP) Evaluation

A gas chromatography/negative-ion chemical ionization tandem mass spectrometry (GC/NICI-MS/MS) was applied to detect the amount of total (free plus esterified) F_2_-IsoPs in seminal plasma. After sample collection, butylated hydroxytoluene (BHT) was added (final concentration 90 *μ*M) and a storage at −80°C was carried out until the assay time. As the first step in F_2_-IsoP detection, a basic hydrolysis was performed by incubation (45°C, 45 min) with 1 N KOH (1 : 0.5, v:v). At the end of the incubation, HCl 1 N was added (1 : 0.5, v : v). Progressively, in each sample, an internal standard (tetradeuterated derivative of prostaglandin F_2*α*_, PGF_2*α*_-d_4_, 500 pg) was added, and two different solid phase extractions (octadecylsilane, C_18_ cartridge and aminopropyl, NH_2_ cartridge) were consecutively carried out. The final eluate was derivatized to convert the carboxyl group of F_2_-IsoPs into pentafluorobenzyl ester and the hydroxyl group into trimethylsilyl ethers [26]. In GC/NICI-MS/MS, *m*/*z* 299 and *m*/*z* 303 ions, derived from the [M-181]^−^ precursor ions of derivatized 15-F_2t_-IsoP (i.e., 8-iso-PGF_2*α*_, the most represented isomer for F_2_-IsoP measurement) and PGF_2*α*_-d_4_, were, respectively, detected.

### 2.7. Statistical Analysis

Data was reported as the median and interquartile range (IQR: 75°-25° centile), and all analyses were performed by SPSS v. 26 (IBM SPSS Statistics). The Kruskal-Wallis test was used to compare the varicocele or idiopathic infertility group and fertile men, followed by, when significant, by Dunn's post hoc test. In order to measure the correlations between the investigated variables, we used Spearman's Rank Correlation Coefficient. A *P* value < 0.05 (two-tailed) was considered statistically significant.

## 3. Results

### 3.1. Semen Characteristics

Semen characteristics and sperm lipid composition of the infertile groups (varicocele or idiopathic infertility) and controls (fertile men) are reported in Table 1. By considering that each sperm sample showed a different number of cells and that in the investigated patients' groups, different amounts of membrane FAs could be present; the absolute values of each investigated parameters were normalized, i.e., expressed as a ratio, to FA contents and sperm parameters (as sperm/ml, sperm apoptosis, necrosis, and immaturity) in order to make them comparable in the investigated groups (Table 2). Fertile men showed a better semen quality compared to that of infertile groups; idiopathic infertile men had a significant decrease in sperm concentration (*P* < 0.01), progressive motility (*P* < 0.05), normal morphology (*P* < 0.01), vitality (*P* < 0.001), and fertility index (*P* < 0.001) and an increase in sperm apoptosis (*P* < 0.05) and sperm necrosis (*P* < 0.05) compared to those observed in the control group.

Fertility index was reduced also in the varicocele group (*P* < 0.05) compared to control. Moreover, in the varicocele group, sperm immaturity percentage was significantly increased compared to that measured in the idiopathic infertility (*P* < 0.05) and fertility (*P* < 0.001) groups.

### 3.2. Seminal and Sperm Lipid Composition

The medians and interquartile ranges of seminal F_2_-IsoP and cPLA_2_ levels and investigated FAs are shown in Table 1.

In the varicocele group, higher levels of F_2_-IsoPs were detected with respect to those of the idiopathic infertility (*P* < 0.01) and fertility (*P* < 0.01) groups. Seminal cPLA_2_ level did not show differences among the investigated groups.

Regarding FA values for SFAs, MUFAs, and n-6 PUFAs, no statistical difference was observed between the control and the patient groups (Table 1).

Total n-3 PUFAs and DHA, one of the n-3 PUFAs, were significantly reduced in idiopathic infertile men compared to controls (*P* < 0.05).

Among the evaluated ratios (Table 2), a significant increase was detected in n-6/n-3 PUFA (*P* < 0.05), F_2_-IsoP/sperm/ml (*P* < 0.01), and immaturity (I)/n-3 PUFA (*P* < 0.05) ratios in both infertile groups as compared to controls. Necrosis (N)/ARA (*P* < 0.05) and N/n-3 PUFAs (*P* < 0.01) increased in idiopathic the infertile group vs. fertile controls. The idiopathic infertile group also showed both a significant increase for the sperm apoptosis (A)/n-3 PUFA ratio, with respect to fertility (*P* < 0.001) and varicocele groups (*P* < 0.05), and an increase for A/n-6 PUFA ratio as compared to the varicocele group (*P* < 0.05).

### 3.3. Relationships between Semen Characteristics and Sperm Fatty Acid Profile or Metabolism

**Figure 1:**
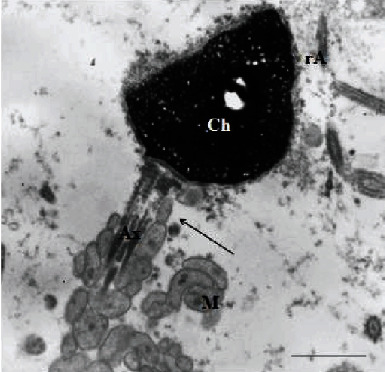
TEM micrograph of a longitudinal section of a necrotic sperm. The plasma membrane appears broken (arrow), the acrosome is reacted (rA), and the chromatin texture (Ch) is altered. The axoneme (Ax) is disrupted, and mitochondria (M) are disassembled and swollen. Bar: 2 *μ*m.

The relationships among the investigated variables in the whole population are shown in Table 3. Oleic acid and total MUFAs correlated negatively with sperm concentration, total sperm number, sperm progressive motility, normal morphology, vitality, and fertility index and positively with sperm necrosis (Figure 1); oleic acid showed also positive correlation with cPLA_2_ levels (*P* < 0.05). Sperm concentration was negatively correlated with margaritic acid (*P* < 0.05) and EPA (*P* < 0.01) content. EPA amount was positively correlated with the percentage of sperm necrosis (*P* < 0.05) and cPLA_2_ level (*P* < 0.01). Sperm vitality was negatively correlated with the SFAs palmitic acid (*P* < 0.05), whereas arachidic acid positively with sperm immaturity (*P* < 0.05).

DHA and total n-3 FAs were positively correlated with sperm concentration, total sperm number, sperm normal morphology, vitality, and fertility index and negatively with sperm necrosis and cPLA_2_.

The amounts of cPLA_2_ were also positively correlated to *trans* 18 : 1 acid level (*P* < 0.05). These results were supported by the relationships between lipids; miristic acid, oleic acid, arachidic acid, EPA, and total MUFAs were positively correlated with each other and negatively correlated with DHA and total n-3 PUFAs. Levels of stearic, palmitoleic, vaccenic, linoleic, eicosadienoic, eicosatrienoic, arachidonic, docosapentaenoic, and *trans* 20 : 1 acids were not correlated to seminal parameters.

In Table 4, the significant correlations between the calculated ratios and sperm characteristics are reported. The EPA/ARA, n-6/n-3 PUFAs, F_2_-IsoPs/ARA, F_2_-IsoPs/sperm/ml, N/ARA, A/ARA, N/n-6, N/n-3, A/n-3, and I/n-3 ratios, except for SFA/MUFA ratio, were negatively correlated with sperm parameters. In addition, EPA/ARA, n-6/n-3 PUFA, and N/n-3 PUFA ratios had positive correlations with cPLA_2_.

Finally, the correlations were performed also in the three analyzed groups (data not shown). In fertile men, palmitoleic acid resulted to be negatively correlated with sperm concentration (*P* < 0.05) and oleic and vaccenic acids with sperm normal morphology and fertility index (both *P* < 0.05). Levels of cPLA_2_ were positively correlated with stearic and margaritic acids (*P* < 0.05). The idiopathic infertility group showed negative correlations between oleic and *trans* 18 : 1 acids with sperm concentration (*P* < 0.01) and progressive motility (*P* < 0.01) and positive correlations between ARA and EPA with sperm necrosis (both *P* < 0.01). Moreover, cPLA_2_ was negatively correlated with DHA and n-3 PUFAs (*P* < 0.05) and positively with EPA (*P* < 0.05). In varicocele, cPLA_2_ was negatively correlated with DHA and n-3 PUFAs (*P* < 0.05) and positively with EPA (*P* < 0.05), *trans* 20: 1 acids and n-6/n-3 PUFA (*P* < 0.05), and EPA/ARA ratios (*P* < 0.01). In the same group, sperm vitality was negatively correlated with palmitoleic acid and total n-6 (*P* < 0.05); sperm apoptosis was positively correlated with the total SFA percentage (*P* < 0.05).

## 4. Discussion

Sperm quality and fertility capability have been related to sperm membrane FAs [10, 15]. In our study, this topic was explored by assessing specific semen features related to the sperm pathologies and fertility index [24] and FA oxidation and metabolism. In particular, sperm parameters were evaluated by light microscopy and sperm ultrastructure was analyzed by TEM. The relationships of sperm characteristics with sperm FA profile, seminal F_2_-IsoP, and cPLA_2_ levels in two groups of infertile patients (idiopathic infertility and varicocele) compared to fertile men were carried out.

As single molecules or as components of molecules, FAs show multiple biological functions involved from the cell membrane composition to energy suppliers and signaling molecules.

The seminal FA lipidome and its connections with sperm characteristics have been investigated in fertile and infertile men [10], and it was suggested that FA profiling may indicate markers of semen quality. Previously, sperm FA content was explored in sperm from patients with abnormal seminal conditions [3, 9, 27] showing that different percentage of FAs was correlated with seminal parameters.

The study of FA profile may individuate candidate markers of semen quality and suggest therapeutic treatments using appropriate FA supplementation [8, 10, 28]. Our study indicated that in the whole fertile and infertile population, oleic acid, total MUFAs, palmitic acid, and cPLA_2_ were linked to elevated levels of sperm necrosis and a reduction of fertility index in addition to a decrease in sperm concentration, progressive motility, normal morphology, and vitality. On the contrary, DHA and total n-3 content appeared strongly positively correlated to a good sperm quality in the total population and in the idiopathic infertile group.

Many studies reported a high concentration of DHA in the spermatozoa of normozoospermic subjects [3, 10, 27, 29]. Recently, it was observed that total n-3 PUFA of normozoospermic semen samples was significantly higher than those from oligozoospermic, asthenozoospermic, and oligoasthenozoospermic individuals [30].

In this study, a relevant characteristic is represented by the fertility index. The fertility index is the number of sperm devoid of defects obtained by TEM evaluation mathematically elaborated [23, 24]. The correlation of the fertility index with the FA content indicates that an abnormal FA metabolism causes spermatogenic dysfunction and consequently may influence male fertility.

The mathematical elaboration of TEM data calculates also the percentage of sperm pathologies such as sperm necrosis, apoptosis, and immaturity in a semen sample [23, 24]. In particular, necrotic sperm are characterized by broken plasma membranes, disrupted chromatin, altered mitochondria, and their percentage increases in the presence of an inflammatory status. It is reported that the membrane destabilization during necrosis is mediated by factors, such as acid-sphingomyelinase, PLA_2_, and calpains. In our research, cPLA_2_ level and sperm necrosis are negatively correlated with DHA and n-3 concentrations suggesting that high levels of cPLA_2_ are linked to an altered sperm and seminal condition. On this point, the membrane destabilization during necrosis is mediated by PLA_2_ [31].

We detected that sperm necrosis and cPLA_2_ were positively linked to palmitoleic and margaritic acids, respectively, and positively correlated to sperm vitality and concentration. This data agrees with that of other authors who detected elevated levels of palmitic, stearic, oleic, linoleic, and arachidonic acids in spermatozoa from patients with altered sperm parameters compared to normozoospermic subjects [32]. The amounts of cPLA_2_ were also negatively linked to normal sperm morphology, and they were increased in infertile varicocele patients [20, 21]. Accordingly, it is well known that cPLA_2_ is involved in inflammatory pathologies and its relationship with oxidative stress and phospholipid impairment has been previously described [33]. The role of cPLA_2_ was also investigated in a drug-induced reproductive impairment model in mice [34], considering that cPLA_2_ influences reproduction, as well as other physiological and pathological processes, by regulating production of prostaglandins, and other lipid mediators from PUFAs [35].

Related to the link between cPLA_2_ and PUFAs, our results showed some significant correlations. The opposite relationships of EPA and DHA with cPLA_2_ are in line with the results showed in depressive disorders where EPA, but not DHA, significantly increased cPLA_2_ gene expression [36]. Since cPLA_2_, besides COX 2, is a key enzyme of the PUFA metabolism, different biological pathways of EPA and DHA could be hypothesized in male infertility, as already reported in other human diseases [36].

Another parameter considered in our study was the level of F_2_-IsoPs, lipid mediators produced from ARA and released in biological fluid by PLA_2_ activity. In varicocele patients, the amount of F_2_-IsoPs was already related to sperm immaturity suggesting an influence of ARA in sperm maturation [19]. This data was confirmed, and the relevance of F_2_-IsoPs in infertility was also reinforced by considering that similar significant data are showed for both F_2_-IsoP absolute value and F_2_-IsoP levels normalized to the ARA content. Moreover, in the infertile varicocele group, palmitoleic acid and total n-6 PUFAs influenced sperm vitality and an increase of SFAs was linked to sperm apoptosis, another sperm pathology evaluated by TEM. Previously, the measurement of SFAs in seminal plasma showed that sperm concentration and motility were negatively correlated with stearic acid and elaidic acid [8, 10, 37]. Recently, in idiopathic infertility patients, a significant positive relationship between F_4_-Neuroprostane level and the percentage of sperm necrosis was observed [38].

An increase of the ratio n-6/n-3 PUFAs was detected in both infertile groups compared to fertile men; a previous study reported a lower seminal n-6/n-3 PUFA ratio in fertile men compared to the infertile ones, probably due to a significantly high amount of total n-3 PUFAs [39]. Safarinejad et al. [40] found higher levels of n-6 PUFAs but lower levels of n-3 PUFAs in the spermatozoa of infertile compared to fertile men.

Interestingly, the ratio EPA/ARA seems to be an index of poor sperm quality by confirming the relevance of an adequate balancing between n-3 and n-6 PUFAs in male fertility. On this issue, a reduced EPA amount with respect to ARA content is influencing inflammatory response [41], which is an event related to male infertility [42]. Considering the SFA/MUFA ratio, it appears to be significantly related to a good quality of sperm, assessed as progressive motility and concentration. This result appears to be linked to the greater presence of FAs without double bonds in their structure (i.e., SFAs), which are less vulnerable to oxidative damage than the FAs containing one double bond (C=C) in their aliphatic chain (i.e., MUFAs) [43]. Accordingly, it is known that the formation of FA radicals increases with increasing unsaturation [44].

The deep knowledge of sperm FA profiles could suggest on personalized nutraceutical treatments to improve male reproduction. Many studies described a semen improvement after n-3 PUFA supplementation in humans [28]; recently, it has been reported that supplemental dietary n-3 PUFAs resulted in an enrichment of membrane FA in rabbit sperm and testes [45].

Although the population investigated in the study can be considered not particularly wide, it is still enough for statistical processing. Moreover, it is important to underline that the strict selection of patients and the multiple investigations carried out on the same seminal sample are not easy to obtain and we believe they can make our study reliable.

## 5. Conclusions

In conclusion, FA composition in sperm membrane and the metabolism of sperm FAs are interrelated parameters, both relevant in sperm maturation processes and fertility.

Our future focus will be to investigate other pathological conditions affecting male fertility and to apply this same protocol in subjects on a controlled diet to explore the effect of dietary FA content in the sperm maturation process.

## Figures and Tables

**Table 1:** Medians and interquartile ranges of semen and sperm characteristics and sperm fatty acid (FA) quantity.

Semen characteristics, sperm fatty acid abundance, and parameters of fatty acid metabolism	Fertility (*n* = 6) Group 1	Idiopathic infertility (*n* = 12) Group 2	Infertile Varicocele (*n* = 12) Group 3	Kruskal-Wallis *P* value	Pairwise comparisons		
					Group 1 vs. group 2	Group 1 vs. group 3	Group 2 vs. group 3
Volume (ml)	4.75 (1.00)	3.75 (2.13)	3.90 (2.00)	0.07			
Sperm/ml × 10^6^	118.50 (116.75)	12.10 (51.95)	53.35 (100.75)	^∗∗^	^∗∗^		
Total sperm number	454.50 (538.95)	49.77 (184.65)	182.00 (220.50)	0.06			
Motility (%)	34.50 (28.75)	17.50 (11.75)	27.50 (21.00)	^∗^	^∗^		
Morphology (%)	17.00 (11.25)	7.00 (4.75)	8.50 (7.50)	^∗∗^	^∗∗^		
Vitality (%)	89.50 (14.25)	50.00 (20.75)	72.50 (20.50)	^∗∗^	^∗∗∗^		
Apoptosis (A) (%)	4.20 (3.18)	8.81 (5.20)	5.82 (2.07)	^∗∗^	^∗^		
Necrosis (N) (%)	22.40 (11.87)	40.65 (16.11)	37.18 (16.65)	^∗^	^∗^		
Immaturity (I) (%)	44.61 (4.53)	53.55 (14.02)	67.71 (12.95)	^∗∗∗^		^∗∗∗^	^∗^
Fertility index (FI)	2907921.50 (16036247.25)	266546.00 (577090.50)	837293.00 (1316138.25)	^∗∗∗^	^∗∗∗^	^∗^	
Seminal F_2_-IsoPs (ng/ml)	8.16 (6.48)	31.12 (22.33)	62.40 (23.08)	^∗∗∗^		^∗∗∗^	^∗∗^
Seminal cPLA_2_ (ng/ml)	0.76 (0.30)	0.86 (2.79)	0.85 (2.23)	0.52			
Myristic acid (%)	0.70 (0.73)	1.00 (0.20)	1.15 (0.45)	0.28			
Palmitic acid (%)	29.85 (12.08)	31.10 (7.95)	28.40 (9.45)	0.48			
Margaritic acid (%)	0.35 (0.18)	0.40 (0.55)	0.35 (0.28)	0.27			
Stearic acid (%)	16.05 (16.70)	15.50 (18.13)	19.55 (15.05)	0.89			
Arachidic acid (%)	0.15 (0.23)	0.50 (0.40)	0.20 (0.55)	0.14			
Total SFAs (%)	52.50 (13.68)	55.05 (14.20)	52.60 (21.95)	0.86			
Palmitoleic acid (%)	0.50 (0.55)	0.90 (1.08)	0.70 (0.85)	0.48			
Oleic acid (%)	5.90 (9.13)	10.45 (7.58)	7.20 (3.95)	0.07			
Vaccenic acid (%)	2.45 (1.90)	3.20 (2.28)	2.70 (1.05)	0.49			
Total MUFAs (%)	8.75 (12.03)	15.65 (8.60)	10.95 (7.48)	0.14			
Linoleic acid (%)	4.10 (2.75)	5.00 (4.43)	5.20 (3.83)	0.76			
Eicosadienoic acid (%)	0.85 (0.20)	0.80 (0.43)	0.75 (0.45)	0.75			
Eicosatrienoic acid (%)	2.85 (3.35)	2.75 (2.83)	4.70 (3.08)	0.11			
Arachidonic acid (%)	3.00 (2.20)	2.65 (2.18)	2.85 (1.98)	0.80			
Total n-6 PUFAs (%)	11.80 (6.00)	10.35 (6.55)	14.65 (9.20)	0.21			
Eicosapentaenoic acid (%)	0.25 (0.23)	0.70 (1.95)	0.10 (1.23)	0.17			
Docosapentaenoic acid (%)	1.05 (0.70)	1.05 (0.58)	0.90 (0.28)	0.60			
Docosahexaenoic acid (%)	24.40 (9.90)	14.55 (10.78)	15.85 (10.88)	^∗^	^∗^		
Total n-3 PUFAs (%)	25.40 (10.38)	17.45 (8.60)	17.75 (8.85)	^∗^	^∗^		
*Trans* 18 : 1 acid (%)	0.00 (0.05)	0.00 (0.10)	0.00 (0.00)	0.30			
*Trans* 20 : 1 acid (%)	0.00 (0.03)	0.00 (0.08)	0.00 (0.00)	0.86			

Volume: volume (ml) of the ejaculate; sperm/ml × 10^6^: sperm concentration in one ml; total sperm number: total number of sperm in the ejaculate; motility: percentage of progressive sperm motility; morphology: percentage of normal sperm morphology assessed with Papanicolaou staining; vitality: percentage of viable sperm by eosin Y; apoptosis: percentage of sperm apoptosis assessed by transmission electron microscopy; necrosis: percentage of sperm necrosis assessed by transmission electron microscopy; immaturity: percentage of sperm immaturity assessed by transmission electron microscopy; fertility index: the number of sperm probably devoid of defects assessed by transmission electron microscopy; F_2_-IsoPs: F_2_-isoprostanes; cPLA_2_: cytoplasmic phospholipase A_2_; total SFAs: sum of saturated fatty acids; total MUFAs: sum of monounsaturated acids; total n-6 PUFAs: sum of omega 6 polyunsaturated fatty acids; total n-3 PUFAs: sum of omega 3 polyunsaturated fatty acids. All FA amounts were reported as relative percentage concentration (%). Statistics are reported (^∗^*P* < 0.05, ^∗∗^*P* < 0.01, and ^∗∗∗^*P* < 0.001).

**Table 2:** Medians and interquartile ranges of evaluated seminal parameters normalized to sperm fatty acid (FA) content or sperm count.

Normalized seminal parameters	Fertility (*n* = 6) Group 1	Idiopathic infertility (*n* = 12) Group 2	Infertile Varicocele (*n* = 12) Group 3	Kruskal-Wallis *P* value	Pairwise comparisons		
					Group 1 vs. group 2	Group 1 vs. group 3	Group 2 vs. group 3
EPA/ARA ratio	0.05 (0.15)	0.30 (0.68)	0.10 (0.30)	0.11			
SFA/MUFA ratio	5.55 (6.95)	3.25 (3.15)	4.80 (3.08)	0.39			
n-6/n-3 PUFA ratio	0.45 (0.23)	0.85 (0.48)	0.75 (0.73)	^∗^	^∗^	^∗^	
F_2_-IsoP/ARA ratio	2.97 (4.36)	10.84 (21.38)	27.07 (21.41)	^∗∗^		^∗∗^	^∗^
F_2_-IsoP/sperm/ml ratio	0.06 (0.10)	1.01 (3.31)	1.05 (2.47)	^∗∗^	^∗∗^	^∗∗^	
N/ARA ratio	7.82 (6.97)	13.90 (8.10)	12.16 (14.71)	^∗^	^∗^		
A/ARA ratio	1.66 (1.50)	3.18 (7.01)	2.53 (2.56)	0.13			
N/n-6 PUFA ratio	1.87 (2.44)	3.94 (1.60)	2.27 (1.45)	^∗^	ns	ns	ns
A/n-6 PUFA ratio	0.40 (0.39)	0.77 (0.69)	0.37 (0.35)	^∗^			∗
I/n-6 PUFA ratio	3.85 (2.90)	5.17 (3.44)	4.07 (2.51)	0.82			
N/n-3 PUFA ratio	0.86 (0.88)	2.83 (1.64)	1.58 (2.01)	^∗∗^	^∗∗^		
A/n-3 PUFA ratio	0.20 (0.17)	0.55 (0.29)	0.32 (0.23)	^∗∗∗^	^∗∗∗^		^∗^
I/n-3 PUFA ratio	1.71 (0.99)	3.06 (4.40)	3.72 (0.80)	^∗^	^∗^	^∗^	

EPA: eicosapentaenoic; ARA: arachidonic acid; SFAs: saturated fatty acids; MUFAs: monounsaturated fatty acids; PUFAs: polyunsaturated fatty acids; F_2_-IsoPs: F_2_-isoprostanes; N: sperm necrosis; A: sperm apoptosis; I: sperm immaturity. Statistics are reported (^∗^*P* < 0.05, ^∗∗^*P* < 0.01, and ^∗∗∗^*P* < 0.001).

**Table 3:** Significant correlations (Spearman's Rho coefficient) between sperm fatty acid (FA) abundance and semen characteristics, seminal isoprostanes (F_2_-IsoPs), and cytoplasmic phospholipase (cPLA_2_) levels.

Sperm fatty acids	Variables positively correlated to the fatty acid	Correlation coefficient (Rho) and statistical significance (*P*)		Variables negatively correlated to the fatty acid	Correlation coefficient (Rho) and statistical significance (*P*)	
		Rho	*P* value		Rho	*P* value
Palmitic acid				Sperm vitality	- 0.362	<0.05
Margaritic acid				Sperm concentration	- 0.422	<0.05
				DHA	- 0.505	<0.01
				Total n-3	- 0.434	<0.05
Arachidic acid	Sperm immaturity	0.428	<0.05			
Oleic acid	Sperm necrosis	0.655	<0.01	Sperm concentration	- 0.561	<0.01
	cPLA_2_	0.414	<0.05	Total sperm number	- 0.377	<0.05
	Total MUFAs	0.968	<0.01	Sperm motility	- 0.599	<0.01
				Sperm morphology	- 0.401	<0.05
				Sperm vitality	- 0.493	<0.01
				Fertility index	- 0.477	<0.01
				DHA	- 0.578 - 0.515	<0.01
				Total n-3		<0.01
Total MUFAs	Sperm necrosis	0.597	<0.01	Sperm concentration	- 0.501	<0.01
	Oleic acid	0.968	<0.01	Sperm motility	- 0.573	<0.01
				Sperm morphology	- 0.372	<0.01
				Sperm vitality	- 0.462	<0.05
				Fertility index	- 0.415	<0.05
				DHA	- 0.483	<0.01
				Total n-3	- 0.414	<0.05
EPA	Sperm necrosis	0.446	<0.05	Sperm concentration	- 0.479	<0.01
	Arachidonic acid	0.471	<0.05	Total sperm number	- 0.398	<0.05
	Oleic acid	0.618	<0.01	DHA	- 0.647	<0.01
	cPLA_2_	0.523	<0.01	Total n-3	- 0.506	<0.01
	Total MUFAs	0.559	<0.01			
	*Trans* 18 : 1	0.603	<0.01			
DHA	Sperm concentration	0.405	<0.05	Sperm necrosis	- 0.576	<0.01
	Total sperm number	0.502	<0.01	cPLA_2_	- 0.518	<0.01
	Sperm morphology	0.580	<0.01	Margaritic acid	- 0.505	<0.01
	Sperm vitality	0.517	<0.01	Oleic acid	- 0.578	<0.01
	Fertility index	0.612	<0.01	Total MUFAs	- 0.483	<0.01
	Total n-3	0.949	<0.01	EPA	- 0.647	<0.01
				*Trans* 18 : 1	- 0.437	<0.05
Total n-3	Sperm concentration	0.475	<0.01	Sperm necrosis	- 0.478	<0.01
	Total sperm number	0.391	<0.05	cPLA_2_	- 0.418	<0.05
	Sperm morphology	0.364	<0.05	Margaritic acid	- 0.434	<0.05
	Sperm vitality	0.470	<0.01	Oleic acid	- 0.515	<0.01
	Fertility index	0.579	<0.01	Total MUFAs	- 0.414	<0.05
	DHA	0.949	<0.01	EPA	- 0.506	<0.01
*Trans* 18 : 1 acid	cPLA_2_	0.371	<0.05	DHA	- 0.437	<0.05
	EPA	0.603	<0.01	Total sperm number	- 0.551	<0.01

Sperm concentration: number of sperm/ml × 10^6^; total sperm number: total number of sperm in the ejaculate; sperm motility: percentage of progressive sperm motility; sperm morphology: percentage of normal sperm morphology assessed with Papanicolaou staining; sperm vitality: percentage of viable sperm by eosin Y; sperm necrosis: percentage of sperm necrosis assessed by transmission electron microscopy; sperm immaturity: percentage of sperm immaturity assessed by transmission electron microscopy; fertility index: the number of sperm probably devoid of defects assessed by transmission electron microscopy; cPLA_2_: cytoplasmic phospholipase seminal levels (ng/ml); total MUFAs: sum of monounsaturated acids; EPA: eicosapentaenoic acid; DHA: docosahexaenoic acid; total n-3: sum of omega 3 fatty acids. All FA amounts were reported as relative percentage concentration (%).

**Table 4:** Significant correlations (Spearman's Rho coefficient) between calculated ratios (parameters normalized to fatty acid (FA) content or sperm count) and semen characteristics, seminal isoprostanes (F_2_-IsoPs), and cytoplasmic phospholipase (cPLA_2_) levels.

Normalized parameters	Variables positively correlated to normalized parameters	Correlation coefficient (Rho) and statistical significance (*P*)		Variables negatively correlated to normalized parameters	Correlation coefficient (Rho) and statistical significance (*P*)	
		Rho	*P* value		Rho	*P* value
EPA/ARA	Sperm necrosis	0.482	<0.01	Sperm concentration	- 0.425	<0.05
	cPLA_2_	0.512	<0.01	Total sperm number	- 0.410	<0.05
	N/ARA	0.526	<0.01	Sperm vitality	- 0.458	<0.05
	N/n-6	0.410	<0.05	SFA/MUFA	- 0.405	<0.05
	A/n-3	0.384	<0.05			
	N/n-3	0.491	<0.01			
SFA/MUFA	Sperm concentration	0.375	<0.05	Sperm necrosis	- 0.504	<0.01
	Sperm motility	0.516	<0.05	EPA/ARA	- 0.405	<0.05
				n-6/n-3	- 0.511	<0.01
n-6/n-3	Sperm necrosis	0.599	<0.01	Sperm concentration	- 0.602	<0.01
	cPLA_2_	0.519	<0.01	Total sperm number	- 0.464	<0.05
	F_2-_IsoPs/sperm concentration	0.616	<0.05	Sperm motility	- 0.551	<0.01
	I/n-3	0.676	<0.01	Sperm morphology	- 0.540	<0.01
	N/n-3	0.741	<0.01	Fertility index	- 0.675	<0.01
				SFA/MUFA	- 0.511	<0.01
F_2-_IsoPs/sperm concentration	Sperm necrosis	0.412	<0.01	Sperm concentration	- 0.833	<0.01
	Sperm immaturity	0.368	<0.05	Sperm morphology	- 0.617	<0.05
	F_2-_IsoPs	0.370	<0.05	Sperm vitality	- 0.370	<0.05
	n-6/n-3	0.616	<0.05	Fertility index	- 0.643	<0.01
	I/n-3	0.558	<0.01			
	N/n-3	0.523	<0.01			
F_2-_IsoPs/ARA	Sperm immaturity	0.522	<0.01	Sperm apoptosis	- 0.499	<0.01
	F_2-_IsoPs	0.854	<0.01			
	N/ARA	0.519	<0.01			
A/ARA	Sperm apoptosis	0.675	<0.01	Sperm vitality	- 0.397	<0.05
	F_2_-IsoPs	0.499	<0.01			
	N/ARA	0.677	<0.01			
	N/n-6	0.392	<0.05			
N/ARA	Sperm necrosis	0.487	<0.01	Sperm vitality	- 0.625	<0.01
	EPA/ARA	0.526	<0.01			
	F_2-_IsoPs/ARA	0.519	<0.01			
	A/ARA	0.677	<0.01			
	N/n-6	0.578	<0.01			
	N/n-3	0.621	<0.01			
N/n-6	Sperm necrosis	0.557	<0.01	Sperm vitality	- 0.638	<0.01
	EPA/ARA	0.410	<0.05			
	A/ARA	0.392	<0.05			
	N/ARA	0.578	<0.01			
	N/n-3	0.481	<0.01			
I/n-3	Sperm necrosis	0.411	<0.05	Sperm concentration	- 0.447	<0.05
	Sperm immaturity	0.530	<0.01	Total sperm number	- 0.390	<0.05
	n-6/n-3	0.676	<0.01	Sperm morphology	- 0.443	<0.05
	F_2-_IsoPs/sperm concentration	0.558	<0.01	Sperm vitality	- 0.389	<0.05
	N/n-3	0.745	<0.01	Fertility index	- 0.507	<0.01
A/n-3	Sperm necrosis	0.413	<0.05	Sperm concentration	- 0.415	<0.05
	Sperm apoptosis	0.666	<0.05	Sperm morphology	- 0.444	<0.01
	EPA/ARA	0.384	<0.05	Sperm vitality	- 0.611	<0.05
	n-6/n-3	0.408	<0.05	Fertility index	- 0.611	<0.01
	F_2-_IsoPs/sperm concentration	0.376	<0.05			
	A/ARA	0.475	<0.05			
	N/ARA	0.362	<0.01			
	N/n-6	0.523	<0.01			
	I/n-3	0.570	<0.01			
N/n-3	Sperm necrosis	0.829	<0.01	Sperm concentration	- 0.594	<0.01
	cPLA_2_	0.449	<0.05	Total sperm number	- 0.458	<0.01
	EPA/ARA	0.491	<0.01	Sperm motility	- 0.468	<0.01
	n-6/n-3	0.741	<0.01	Sperm morphology	- 0.467	<0.01
	F_2-_IsoPs/sperm concentration	0.523	<0.01	Sperm vitality	- 0.643	<0.01
	N/ARA	0.621	<0.01	Fertility index	- 0.692	<0.01
	N/n-6	0.481	<0.01			
	I/n-3	0.745	<0.01			
	A/n-3	0.653	<0.01			

Sperm concentration: number of sperm/ml × 10^6^; total sperm number: total number of sperm in the ejaculate; sperm motility: percentage of progressive sperm motility; sperm morphology: percentage of normal sperm morphology assessed with Papanicolaou staining; sperm vitality: percentage of viable sperm; sperm apoptosis: percentage of sperm apoptosis assessed by transmission electron microscopy; sperm necrosis: percentage of sperm necrosis assessed by transmission electron microscopy; sperm immaturity: percentage of sperm immaturity assessed by transmission electron microscopy; fertility index: the number of sperm probably devoid of defects assessed by transmission electron microscopy; F_2_-IsoPs: F_2_-isoprostane seminal levels (ng/ml); cPLA_2_: phospholipase seminal levels (ng/ml); EPA: eicosapentaenoic acid; A: sperm apoptosis; ARA: arachidonic acid; N: sperm necrosis; SFAs: saturated fatty acids; MUFAs: monounsaturated fatty acids; n-3: sum of omega 3 FAs; n-6: sum of omega 6 FAs. All FA amounts were reported as relative percentage concentration (%).

## Data Availability

The data used to support the findings of this study is available from the corresponding author upon request.
